# Anion exchange coupled with the reduction and dimerisation of a copper(ii) nitrate complex of tripyridyl dithioether *via* a single-crystal-to-single-crystal transformation†
†Electronic supplementary information (ESI) available: PXRD patterns, ESI-MS spectrum, SEM images, AFM images, UV spectra, crystal structures and TGA data. CCDC 1511571–1511577. For ESI and crystallographic data in CIF or other electronic format see DOI: 10.1039/c6sc05341f



**DOI:** 10.1039/c6sc05341f

**Published:** 2017-01-03

**Authors:** Hyeong-Hwan Lee, In-Hyeok Park, Seulgi Kim, Eunji Lee, Huiyeong Ju, Jong Hwa Jung, Mari Ikeda, Yoichi Habata, Shim Sung Lee

**Affiliations:** a Department of Chemistry , Research Institute of Natural Science , Gyeongsang National University , Jinju 52828 , South Korea . Email: pihghost@nate.com ; Email: sslee@gnu.ac.kr ; Tel: +82 55-772-1483; b Education Center , Faculty of Engineering , Chiba Institute of Technology , 2-1-1 Shibazono , Narashino , Chiba 275-0023 , Japan; c Department of Chemistry , Toho University , 2-2-1 Miyama , Funabashi , Chiba 274-8510 , Japan

## Abstract

It is a challenge to develop methodologies involving multiple transformations for preparing new materials that cannot be obtained *via* direct synthesis. Herein, we report an anion exchange process accompanying cation reduction and dimerisation *via* a single-crystal-to-single-crystal transformation. First, a direct reaction of the flexible tripyridyl dithioether ligand **L** with CuI afforded a mixture of four bis(ligand) complexes (**1a–1d**). To avoid the formation of undesired mixed products, a copper(ii) nitrate complex-mediated approach involving anion exchange and cation reduction was employed to generate a monomeric complex, [Cu^II^(**L**)NO_3_]NO_3_·toluene (**2**). When the dark blue crystals of **2** were immersed in an aqueous NaI solution, the crystals were transformed to a pale yellow dimeric copper(i) iodide complex, [(μ-Cu^I^_2_I_2_)(**L**)_2_] (**3**). The observed anion exchange promotes the reduction of copper(ii) to copper(i) at the expense of I^–^/I_3_^–^ oxidation as well as dimerisation *via* the formation of a Cu_2_I_2_ cluster. This result corresponds to the synthesis of a compound that otherwise was not able to be prepared *via* a direct synthetic procedure.

## Abstract 2

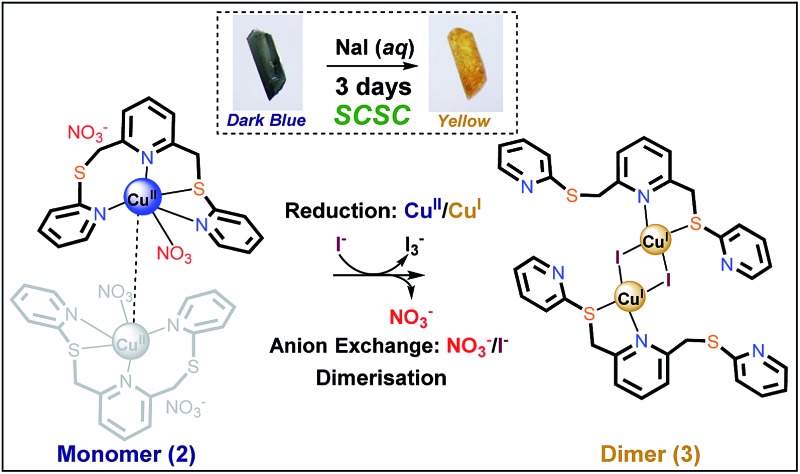
We report that the anion exchange induced conversion of the oxidation state of the central metal and the dimerisation in the complex *via* an SCSC transformation.

## Introduction

Post-synthetic modification (PSM) or post-assembly modification (PAM) *via* a single-crystal-to-single-crystal (SCSC) transformation has proven to be a powerful tool, not only for creating new materials, but also for understanding the mechanistic details of their formation.^1^ Hence, the construction of extended solid materials *via* an external exchange process (such as an SCSC guest-exchange) has been of considerable interest because it can result in translocation/exchange of atoms and ions, in particular in solid matrices.^2^

The PSM approach involving multiple molecular processes offers a relatively unexplored field for obtaining novel materials. However, in some cases anion exchange has been shown to be accompanied by bond breaking and/or bond making. The study of this behaviour involving metal-anion coordination has become an important emerging field due to its relevance to biological processes, environmental pollution, ionic liquids, catalysis, lithium batteries and health related areas.^3^ On the other hand, the SCSC transformations, involving the chemical reduction of a metal centre, provide a potentially important modification approach for generating new materials.^4^

**Fig. 1:**
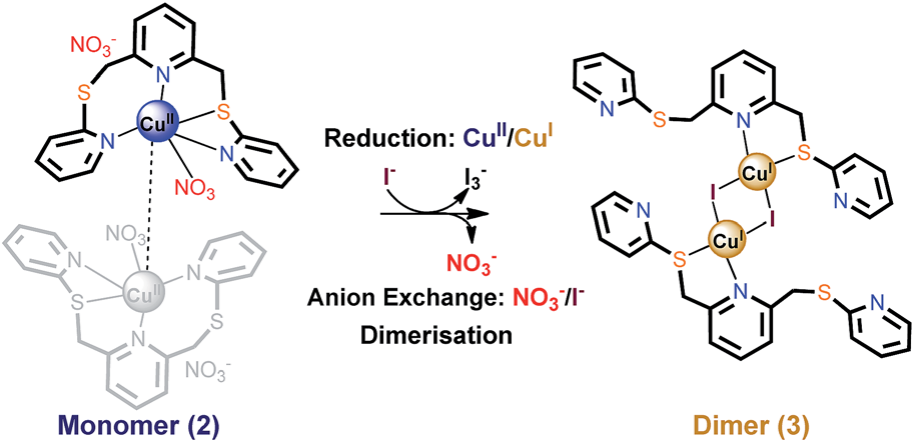
Anion exchange of copper(ii) nitrate complex accompanying reduction and dimerisation *via* a SCSC transformation.

In our recent study, direct reaction of tripyridyl dithioether [**L**, 2,6-bis(2-pyridylsulfanylmethyl)pyridine, Fig. 1] with copper(i) iodide was shown to form a mixture of four complexes that reflects the flexible nature of the ligand (see later). To avoid the formation of these mixed products, we have investigated a system that involves simultaneous anion exchange coupled with the reduction of a metal centre, as depicted in Fig. 1. The observed process appears to be the first example of the simultaneous exchange of an anion species and change of the metal oxidation state of the central metal in a coordination compound accompanying an SCSC transformation.

## Results and discussion

**Fig. 2:**
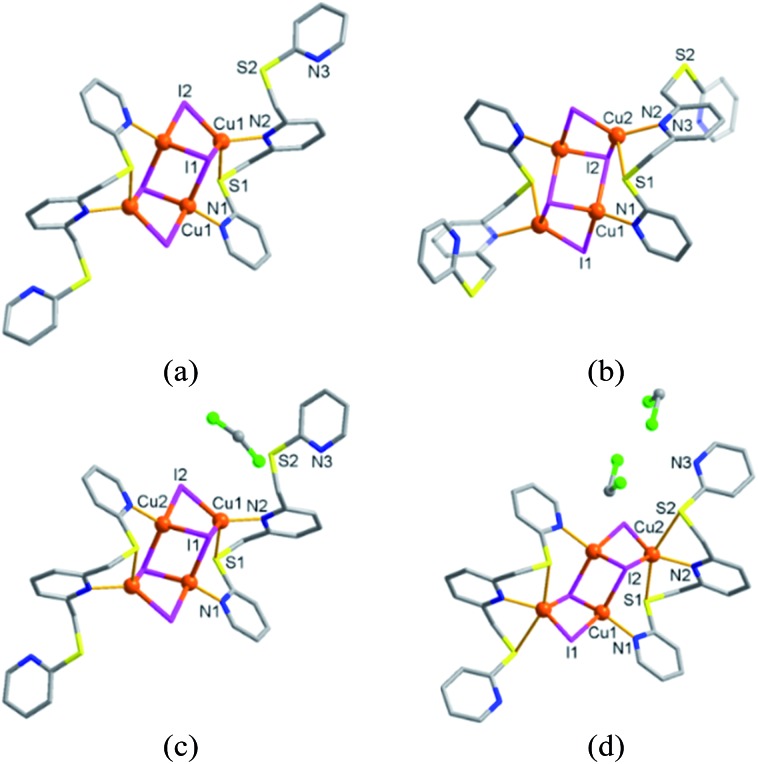
Crystal structures of (a) **1a**, [(Cu_4_I_4_)(**L**)_2_], (b) **1b**, [(Cu_4_I_4_)(**L**)_2_], (c) **1c**, [(Cu_4_I_4_)(**L**)_2_]·CH_2_Cl_2_, and (d) **1d**, [(Cu_4_I_4_)(**L**)_2_]·2CH_2_Cl_2_.

Reaction of the required precursor thiol and ditosylate in a 2 : 1 molar ratio in the presence of potassium carbonate under reflux resulted in the formation of **L** *via* C–S bond formation (yield, 80%; Fig. S1†). The procedure employed in the present study showed improved yields over the related literature method for **L**.^5^ The reaction of **L** with copper(i) iodide to yield the mixture of four crystalline products (**1a–1d** in Fig. 2, S2 and S3†) was carried out in acetonitrile/dichloromethane. Single crystal X-ray diffraction (SC-XRD) analyses confirm that **1a–1d** have similar structures, each adopting a bis(ligand) complex arrangement that is linked by a stepped cubane cluster (Cu_4_I_4_) to yield [(Cu_4_I_4_)(**L**)_2_] (**1a**), [(Cu_4_I_4_)(**L**)_2_] (**1b**), [(Cu_4_I_4_)(**L**)_2_]·CH_2_Cl_2_ (**1c**), and [(Cu_4_I_4_)(**L**)_2_]·2CH_2_Cl_2_ (**1d**). The metal complex units in **1a–1d** are composed of identical building blocks; namely, two **L** ligands and one bridging Cu_4_I_4_ cluster with an ‘open’ cubane form. Thus, the overall conformational differences in **1a–1d** mainly originate from the presence of solvent and changes in the coordinated ligand conformation (Fig. S3–S9†). Further, in **1a–1c**, each **L** is tridentate, whereas it is tetradentate in **1d**.

**Fig. 3:**
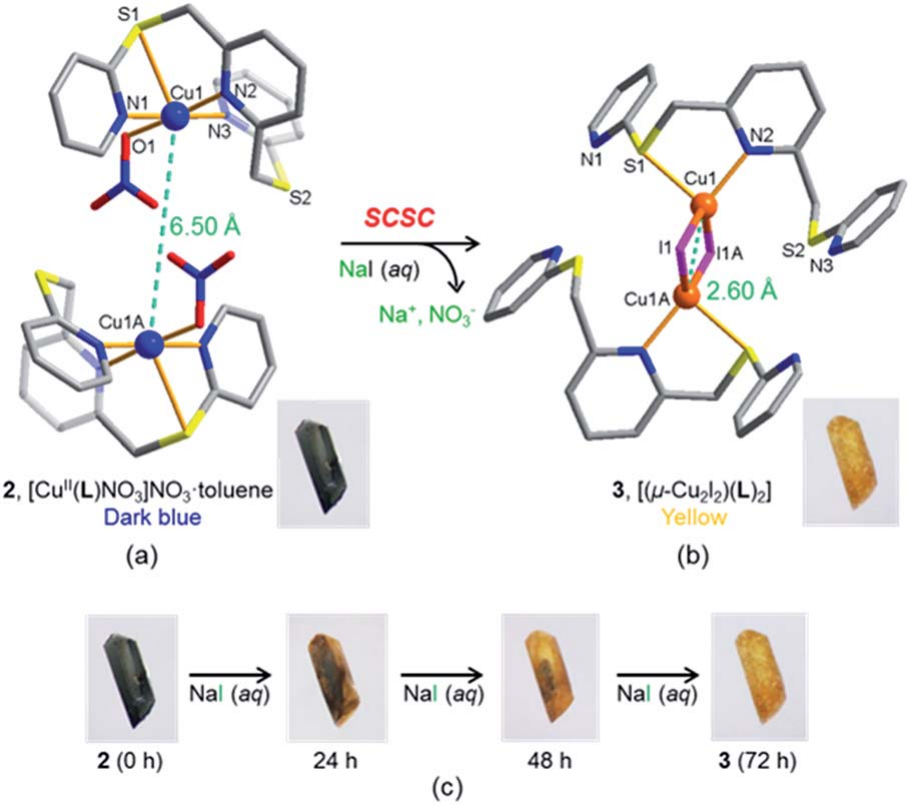
Anion-induced conversion of (a) **2**, [Cu^II^(**L**)NO_3_]NO_3_·toluene to (b) **3**, [(μ-Cu^I^_2_I_2_)(**L**)_2_] accompanied by copper reduction (from 2^+^ to 1^+^) and dimerisation *via* SCSC transformation in 3 M NaI aqueous solution. (c) The gradual colour changes of a dark blue single crystal of **2** to a yellow single crystal of **3** in a 3 M NaI aqueous solution.

As an alternative, a Cu(ii) complex-mediated two-step approach was employed. In the first step, copper(ii) nitrate was reacted with **L** in acetonitrile/dichloromethane to yield the dark blue crystalline product **2** (Fig. 3a). SC-XRD analysis revealed that **2** is a typical mononuclear copper(ii) complex of type [Cu^II^(**L**)NO_3_]NO_3_·toluene, in which one nitrate ion coordinates, whereas the other resides in the lattice together with the toluene molecule. Complex **2** crystallised in the monoclinic space group *C*2/*c* with *Z* = 8. The copper(ii) centre is in a pentacoordinated N_3_OS environment and can be best described as a square-pyramidal geometry (*τ* value: 0.02),^6^ with three pyridyl N atoms from **L** and one O atom from nitrate in the square plane, with the apical position occupied by a S atom from **L**. The Cu1–S1 distance is elongated at 2.690 Å (Fig. S10 and S11†). The experimental and simulated powder X-ray diffraction (PXRD) patterns for **2** confirmed that this product is homogeneous (Fig. S12 and S13†).

When the dark blue single crystals of **2** were immersed in a 3 M aqueous solution of NaI for four days, the size and shape of the daughter crystals **3** retained those of **2**. While retention of single crystallinity was maintained, the colour changed from dark blue (**2**) to pale yellow (**3**, see Fig. 3c). Surprisingly, the SC-XRD analysis revealed that **3** is a bis(ligand) copper(i) iodide complex of type [(μ-Cu^I^_2_I_2_)(**L**)_2_] in which two Cu(i) centres are bridged by two I^–^ ions.

Unlike its precursor **2**, a perspective view of **3** (Fig. 3b) shows that each copper(i) centre is four-coordinated, being bound to one pyridine N atom and one S atom from one **L** ligand. The two remaining sites are occupied by two iodide ions giving rise to a distorted tetrahedral geometry. The most striking structural feature of **3** is its dimeric form linked by a Cu^I^_2_I_2_ square cluster. The associated change in the Cu1···Cu1A distance from 6.50 Å to 2.60 Å in going from **2** to **3** is remarkable in the solid state.

Note that the anion-induced SCSC transformation from **2** to **3** involves the rearrangement of the coordination sphere as well as framework distortion with, as mentioned already, a redox process associated with this change. Indeed, only a limited number of examples are reported where a complete structural change on anion exchange occurs in the solid state.^7^ In these previous cases, the structural conversion mainly involved bond breaking and/or bond making of the ligand/anion bond, without significant changes in the location of the metal centres and ligands.

When the solid copper(ii) nitrate complex **2** is immersed in 3 M sodium iodide aqueous solution, it is proposed that the reaction presented in eqn (1) takes place. The nitrate ions in **2** are replaced by I^–^ ions to generate both CuI and I_2_, with the reduced copper(i) iodide aggregating to afford a Cu_2_I_2_ unit. In the presence of surplus I^–^, I_2_ will be converted to I_3_^–^.^8^ The liberated I^–^, I_2_, and I_3_^–^ were monitored by ESI-mass spectrometry (Fig. S14†).1
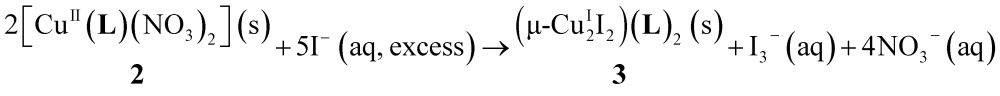



In **3**, the individual monomeric complex units are connected through Cu–(I)_2_–Cu bonds, to form its dimeric structure. The structural change involves the conversion of monomer to dimer and the reduction of the coordination number from five to four at each copper centre. Note that the approach employed is also influenced by the match between the hardness or softness of the anion (Lewis base) and the metal centre (Lewis acid). That is, upon exposure to an aqueous solution of NaI, the relatively hard Cu^2+^ Lewis acid is reduced to soft Cu^+^, and then the hard base NO_3_^–^ ion is easily exchanged by the soft I^–^ ion.

**Fig. 4:**
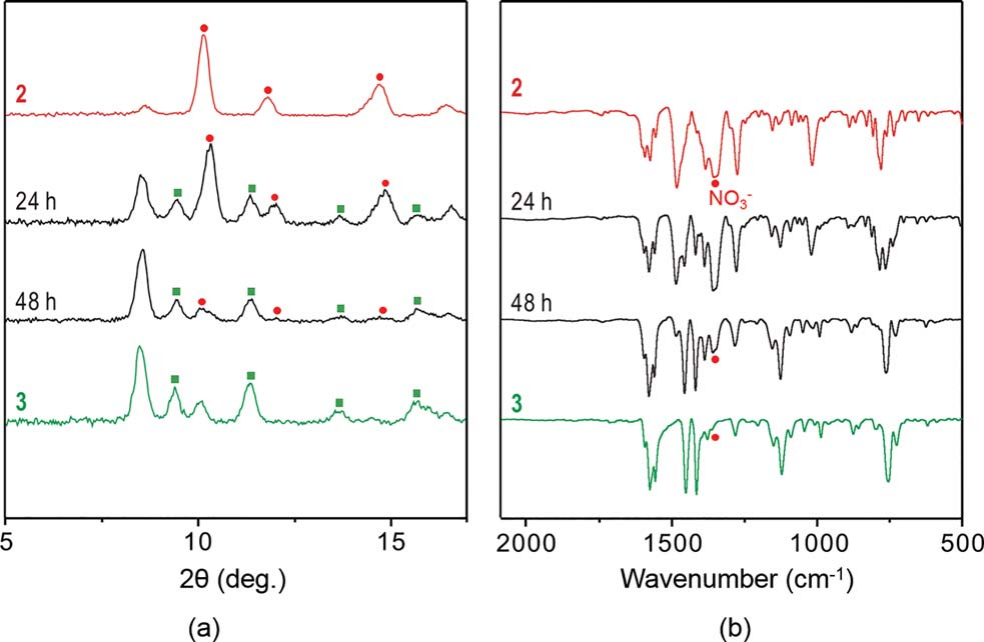
(a) PXRD patterns and (b) IR spectra of **2** (top) before and (bottom) after anion exchange employing 3 M NaI aqueous solution.

The experimental and simulated PXRD patterns of **3** confirmed its homogeneous nature (Fig. S8†), indicating that complete SCSC transformation had occurred. PXRD patterns and IR spectra were obtained for the same samples before and after anion exchange in the solid state (Fig. 4). Both results are in agreement that the complete anion exchange has occurred. The elemental mapping by the energy dispersive spectroscopy (EDS) with SEM for both compounds also shows that **3** contains I atom as one component (Fig. S15†).

**Fig. 5:**
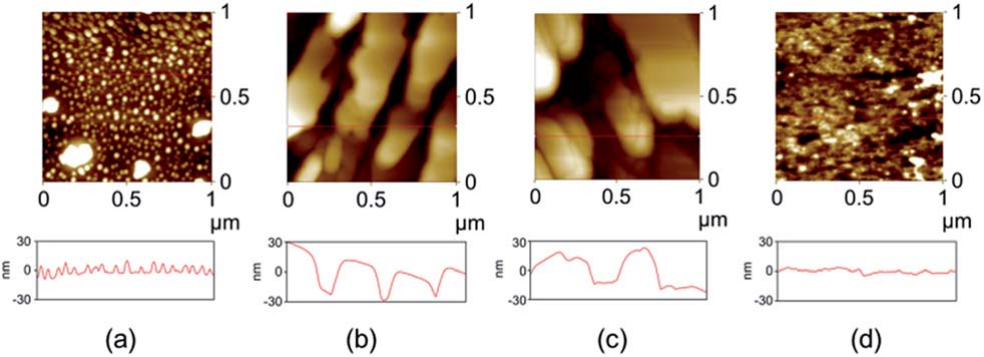
AFM images and height profiles of the surface of a single crystal of **2** before and after immersing in a 3 M NaI aqueous solution: (a) before anion exchange, (b) 24 h, (c) 48 h, and (d) 72 h.

To investigate any possible crystal morphology and other variation, single crystal samples before and after anion exchange were used for atomic force microscopy (AFM) investigations. The results indicate that the crystal surface profile undergoes significant alteration, implying a restructuring of the crystal surface (Fig. 5 and S16†). After 24 and 48 h, for example, the relatively homogenous and flat crystal surface of the original crystal (Fig. 5a) becomes rough, showing holes and clefts (Fig. 5b and c). However, after 72 h, the surface of the anion exchanged sample had become more regularly ordered and consisted of microcrystallites (Fig. 5d). These observations are typical for a solvent-mediated transformation,^9^ during which a new crystalline phase is generated on the surface.

**Fig. 6:**
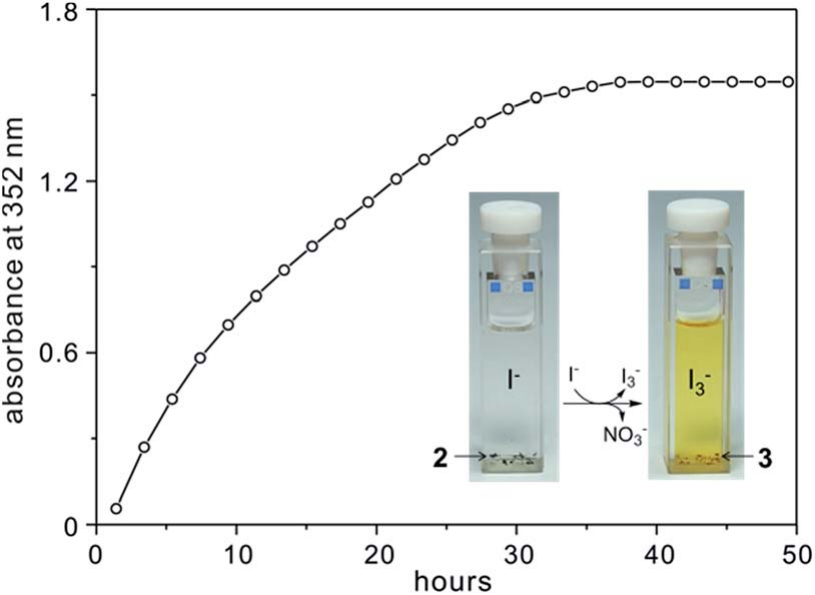
Time-dependent profile for NO_3_^–^ release from **2** by detecting I_3_^–^ in the supernatant NaI aqueous solution.

The anion exchange process was monitored by UV-vis spectroscopy at intervals based on the maximum adsorption peak of I_3_^–^ at 352 nm.^10^ As shown in Fig. 6 and S17,† several single crystals of **2** plus 3 M NaI aqueous solution (3 mL) were added to a standard (1 cm) UV-vis quartz cell. Subsequently, the mother solution of the sample in the cell slowly changed from colourless to light yellow/brown (see the images in Fig. 6), implying the formation of I_3_^–^, as shown in eqn (1). The absorbance due to I_3_^–^ increases with time until it reaches a maximum (36 h) where the time-dependent plot levels out.

In ‘control’ experiments, we found that no anion exchange or reduction occurred when we attempted the same procedure in aqueous NaCl or NaBr solution. Instead, when the dark blue single crystals of the mononuclear copper(ii) nitrate complex **2** were exposed to 3 M NaCl aqueous solution or distilled water, on careful observation, the dark blue colour was seen to gradually disappear, finally turning colourless, leading to the recovery of single crystals of **L** (Fig. S18–S20†). In the TGA data, **3** shows a higher thermal stability than **2**, which decomposes around 130 °C after the loss of the lattice solvent (Fig. S21†).

## Conclusions

As far as we are aware, the present study is the first example of an anion exchange process coupled with the reduction and dimerisation of a metal centre of a complex occurring during an SCSC transformation. However, it is also clear that more work is required to understand the fundamental aspects governing the unique multiple transformation observed in this study. It is expected that this facile approach might provide a promising and efficient post-synthetic strategy for practical use in the future.
