# Supplementary material for: Anion exchange coupled with the reduction and dimerisation of a copper(ii) nitrate complex of tripyridyl dithioether via a single-crystal-to-single-crystal transformation
Source: Chem Sci. 2017 Jan 3;8(4):2592–6. doi: 10.1039/c6sc05341f (PMC5431679; doi:10.1039/c6sc05341f)
Supplement: Supplementary file 1 [file SC-008-C6SC05341F-s001.pdf]

Electronic Supplementary Material (ESI) for Chemical Science.
This journal is © The Royal Society of Chemistry 2017

Electronic Supplementary Information
for

Anion exchange coupled with reduction and dimerisation of a copper(II) nitrate complex of tripyridyl dithioether via a single-crystal-to-single-crystal transformation

Hyeong-Hwan Lee,[a] In-Hyeok Park,*[a] Seulgi Kim,[a] Eunji Lee,[a] Huiyeong Ju,[a] Jong Hwa Jung,[a] Mari Ikeda,[b] Yoichi Habata[c] and Shim Sung Lee*[a]

[a] Department of Chemistry and Research Institute of Natural Science, Gyeongsang National University, Jinju 52828, S. Korea

[b] Education Center, Faculty of Engineering, Chiba Institute of Technology, 2-1-1 Shibazono, Narashino, Chiba 275-0023, Japan

[c] Department of Chemistry, Toho University, 2-2-1 Miyama, Funabashi, Chiba 274-8510, Japan

Experimental section

Preparation of $[(Cu_4I_4)(L)_2]$ (1a), $[(Cu_4I_4)(L)_2]$ (1b), $[(Cu_4I_4)(L)_2]{\cdot}CH_2Cl_2$ (1c) and $[(Cu_4I_4)(L)_2]{\cdot}2CH_2Cl_2$ (1d). A small amount of toluene was added to a dichloromethane (1 mL) solution of **L** (Mp: 95-96 °C, 30.0 mg, 0.092 mmol); then the required copper(I) iodide (17.6 mg, 0.092 mmol) in acetonitrile was layered on the toluene phase; the (layered) mixture of four species **1a-1d** afforded a pale yellow crystalline products suitable for X-ray analysis.

1a: Mp: 201-203 °C (decomp.). IR (KBr pellet): 3055, 2984, 2893, 1593, 1577, 1557, 1453, 1413, 1396, 1281, 1154, 1119, 878, 754 cm^{-1}. Anal. Calcd $[C_{34}H_{30}N_6S_4Cu_4I_4]$: C, 28.91; H, 2.14; N, 5.95; S, 9.08. Found: C, 29.06; H, 2.13; N, 5.93; S, 9.25%. Mass spectrum *m/z* (ESI): 388.3 $[Cu(\mathbf{L})]^+$.

1b: Mp: 187-190 °C. IR (KBr pellet): 3056, 2938, 2903, 1577, 1557, 1453, 1415, 1363, 1280, 1147, 1122, 1052, 751, 720 cm^{-1}. Anal. Calcd for $[C_{34}H_{30}N_6S_4Cu_4I_4]$: C, 28.91; H, 2.14; N, 5.95; S, 9.08. Found: C, 29.01; H, 2.08; N, 5.94; S, 9.39%. Mass spectrum *m/z* (ESI): 388.2 $[Cu(\mathbf{L})]^+$.

1c: Mp: 198-201 °C. IR (KBr pellet): 3045, 2971, 2898, 1596, 1575, 1556, 1453, 1414, 1281, 1214, 1153, 1118, 879, 757 cm^{-1}. Anal. Calcd for $[C_{34}H_{30}N_6S_4Cu_4I_4]$: C, 28.91; H, 2.14; N, 5.95; S, 9.08. Found: C, 29.15; H, 2.09; N, 5.97; S, 9.35%. Mass spectrum *m/z* (ESI): 388.2 $[Cu(\mathbf{L})]^+$.

1d: Mp: 192-194 °C. IR (KBr pellet): 3057, 2956, 2898, 1577, 1557, 1453, 1415, 1282, 1214, 1156, 1121, 1052, 879, 755 cm^{-1}. Anal. Calcd for $[C_{34}H_{30}N_6S_4Cu_4I_4]$: C, 28.91; H, 2.14; N, 5.95; S, 9.08. Found: C, 28.84; H, 2.15; N, 5.85; S, 8.81%. Mass spectrum *m/z* (ESI): 388.2 $[Cu(\mathbf{L})]^+$.

Preparation of $[Cu(L)NO_3](NO_3){\cdot}$toluene (2). A small amount of toluene was added to a dichloromethane (1 mL) solution of **L** (30.1 mg, 0.092 mmol); then the required copper(II) nitrate (22.3 mg, 0.092 mmol) in acetonitrile was layered on the toluene phase; the (layered) mixture afforded a dark blue crystalline product suitable for X-ray analysis. Mp: 140-141 °C (decomp.). IR (KBr pellet): 3087, 3019, 2944, 2911, 1592, 1482, 1384, 1354 (NO_3^-), 1274, 1153, 1016, 888, 807, 735 cm^{-1}. Anal. Calcd for $[C_{20.5}H_{19}N_5O_6S_2Cu]$:

C, 44.04; H, 3.43; N, 12.53; S, 11.47. Found: C, 43.89; H, 3.35; N, 12.62; S, 11.44%.

Preparation of [(Cu_2I_2)(L)$_2$] (3). Freshly prepared single crystals of **2** were added to 3 M NaI aqueous solution (2 mL) for four days. Compound **3** was isolated by washing with water. Mp: 128-131 °C. IR (KBr pellet): 3045, 2995, 2975, 1575, 1556, 1451, 1414, 1376, 1280, 1149, 1121, 986, 875, 754 cm^{-1}; Anal. Calcd for [$C_{34}H_{38}N_6O_4S_4Cu_2I_2$]: C, 39.58; H, 2.93; N, 8.15; S, 12.43. Found: C, 39.69; H, 3.03; N, 8.29; S, 12.67%. Mass spectrum *m/z* (FAB): 1030.9 [(Cu_2I_2)(**L**)$_2$ + H]$^+$.

X-ray crystallographic analysis. Crystal data for **1a-d**, **2** and **3** were collected on a Bruker SMART APEX II ULTRA diffractometer equipped with graphite monochromated Mo Kα radiation (λ = 0.71073 Å) generated by a rotating anode. Data collection, data reduction, and semi-empirical absorption correction were carried out using the software package of APEX2.[15] Calculations for the structure determinations were carried out using the SHELXTL package.[16] Since the lattice solvent molecules in **2** are highly disordered, the contribution of solvent electron density was removed by the SQUEEZE routine in PLATON.[S1] The relevant crystal data collection and refinement data for the crystal structures of **1a-1d**, **2**, **3** and **L** are summarised in Tables S1 and S2. CCDC 1511571 (**1a**), 1511572 (**1b**), 1511573 (**1c**), 1511574 (**1d**), 1511575 (**2**), 1511576 (**3**) and 1511577 (**L**) contain the supplementary crystallographic data for this paper. These data can be obtained free of charge from The Cambridge Crystallographic Data Centre via www.ccdc.cam.ac.uk/data_request/cif.

Reference

S1. A. L. Spek, PLATON, A Multipurpose Crystallographic Tool, University of Ultrecht, Ultrecht, The Netherlands, 2003.

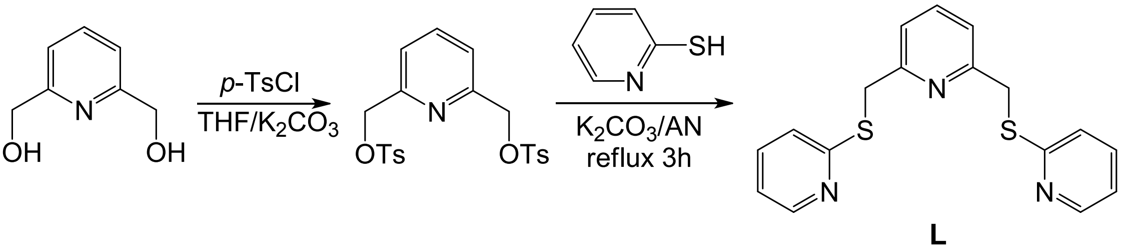

L

Fig. S1 Synthesis of **L**.

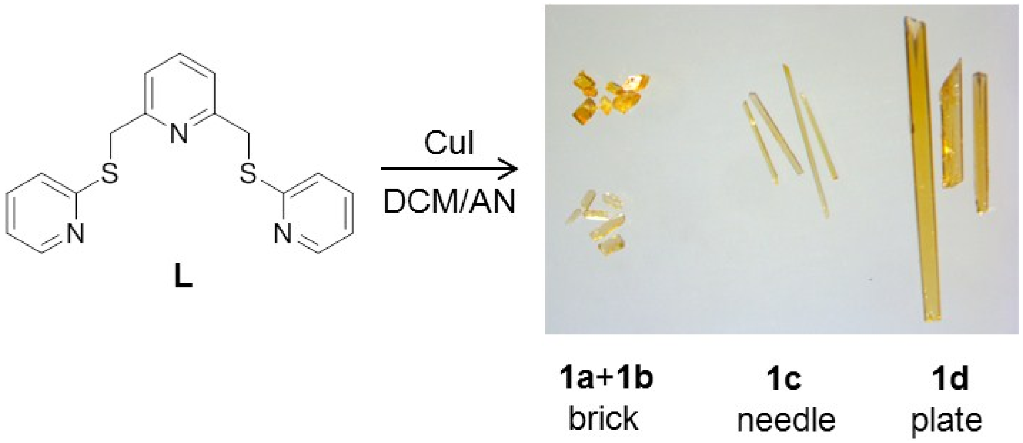

Fig. S2 Photomicroscope image of a mixture of products (**1a**-**1d**).

Detailed structural descriptions of 1a-d: Since the yellow product shows three crystal morphologies (or crystal habits), it was possible to manually separate three types (brick, needle, and long plate) under the microscope. The powder X-ray diffraction (PXRD) patterns and single-crystal X-ray analysis of each type revealed that the observed difference in the crystal habit meant different species except for the brick-shaped ones. In this case, two species (**1a** and **1b**) were identified by cell parameter determination. As a result, a combination of the naked eye [needle (**1c**) and long plate (**1d**) crystals] and the cell parameter determinations [brick crystals (**1a** and **1b**)] allowed the identification (and separation) of four species form the mixed product.

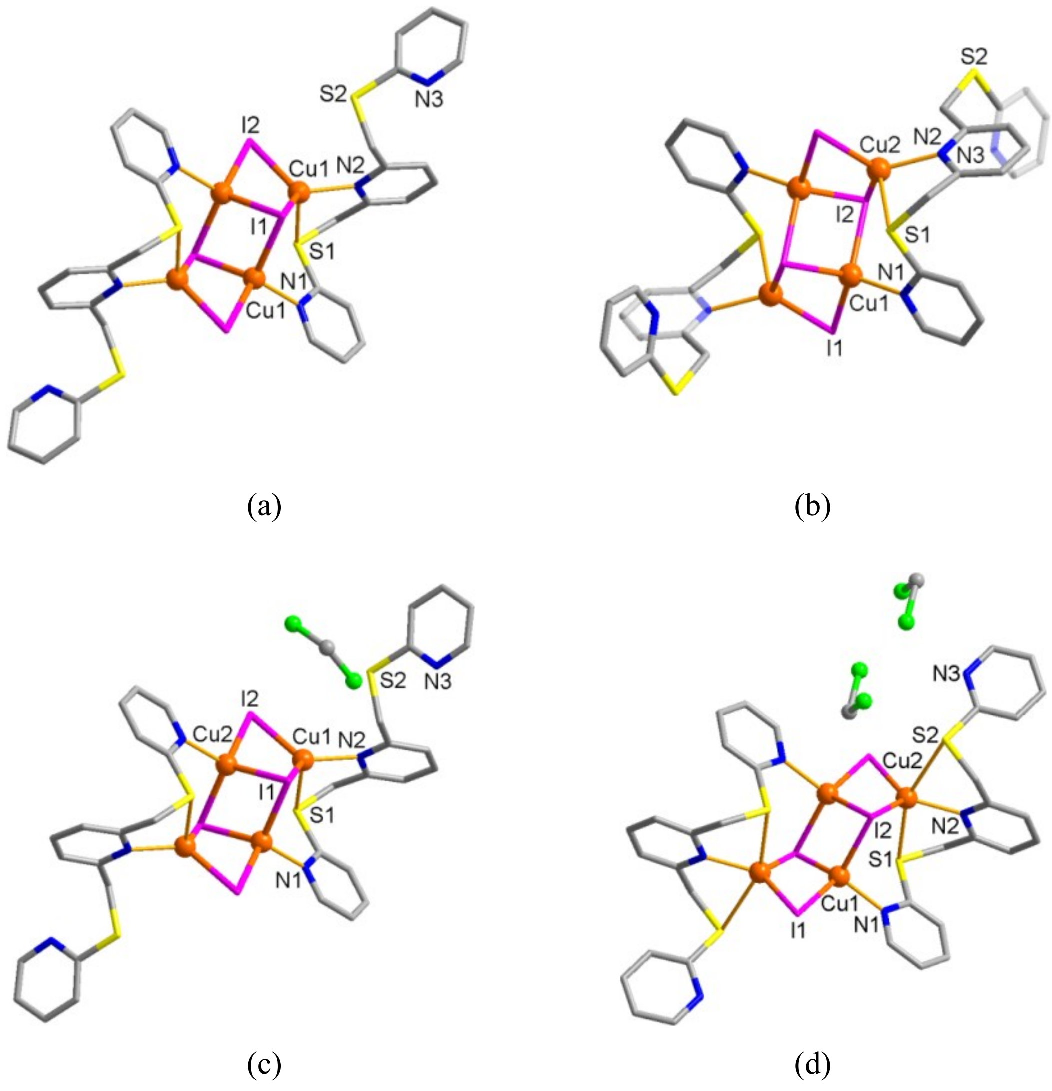

Fig. S3 Crystal structures of (a) **1a**, $[(Cu_4I_4)(\mathbf{L})_2]$, (b) **1b**, $[(Cu_4I_4)(\mathbf{L})_2]$, (c) **1c**, $[(Cu_4I_4)(\mathbf{L})_2]\cdot CH_2Cl_2$ and (d) **1d**, $[(Cu_4I_4)(\mathbf{L})_2]\cdot 2CH_2Cl_2$.

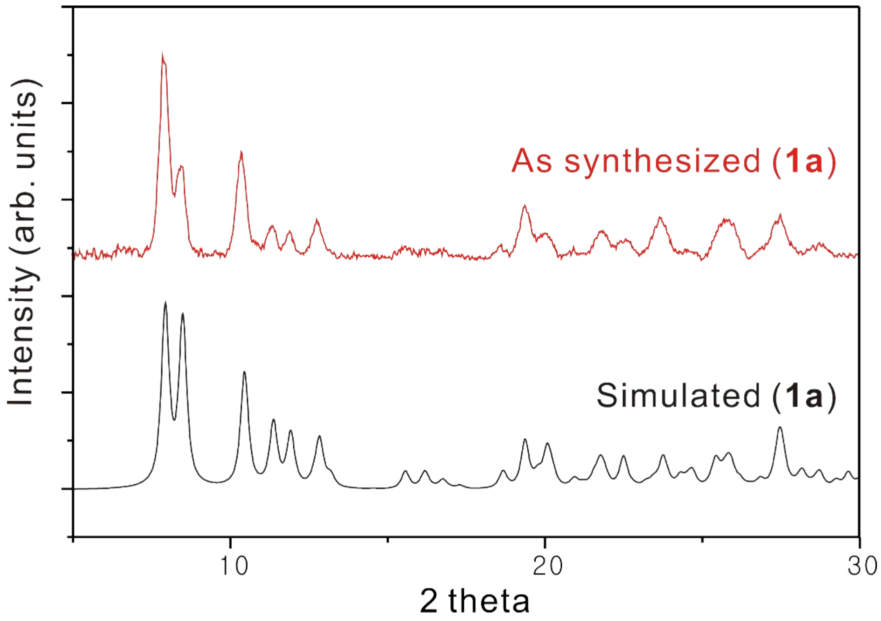

Fig. S4 PXRD patterns for **1a**: (top) as synthesized and (bottom) simulated from the single crystal X-ray data.

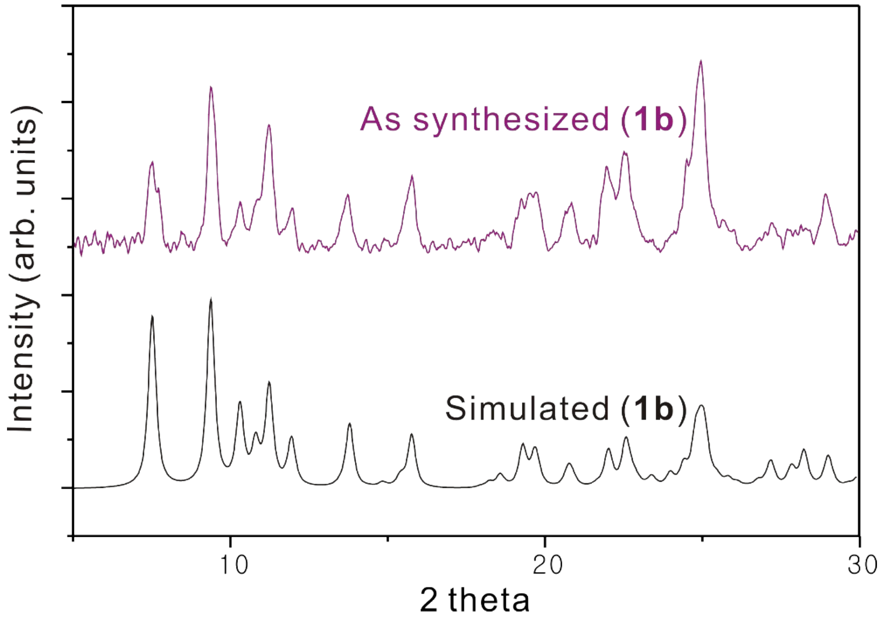

Fig. S5 PXRD patterns for **1b**: (top) as synthesized and (bottom) simulated from the single crystal X-ray data.

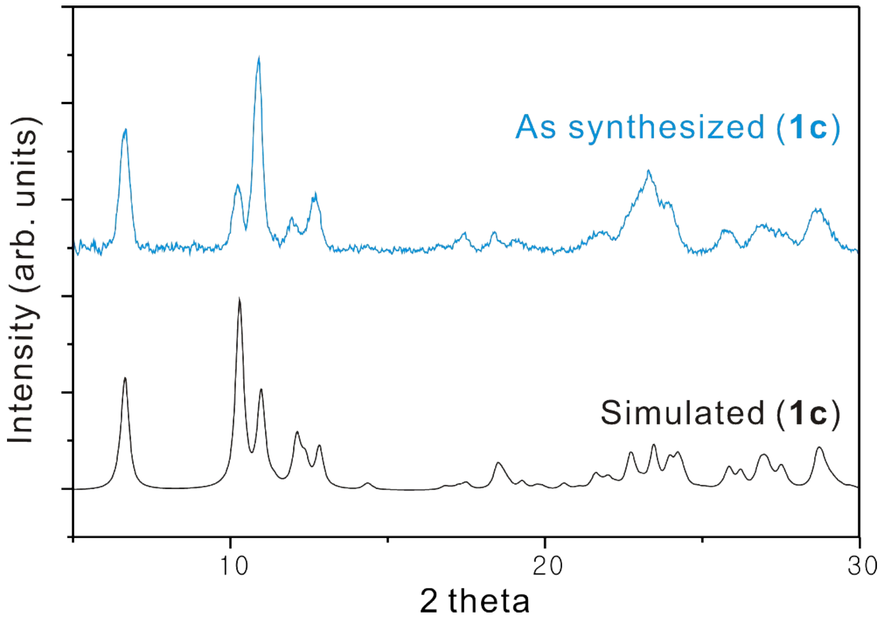

Fig. S6 PXRD patterns for **1c**: (top) as synthesized and (bottom) simulated from the single crystal X-ray data.

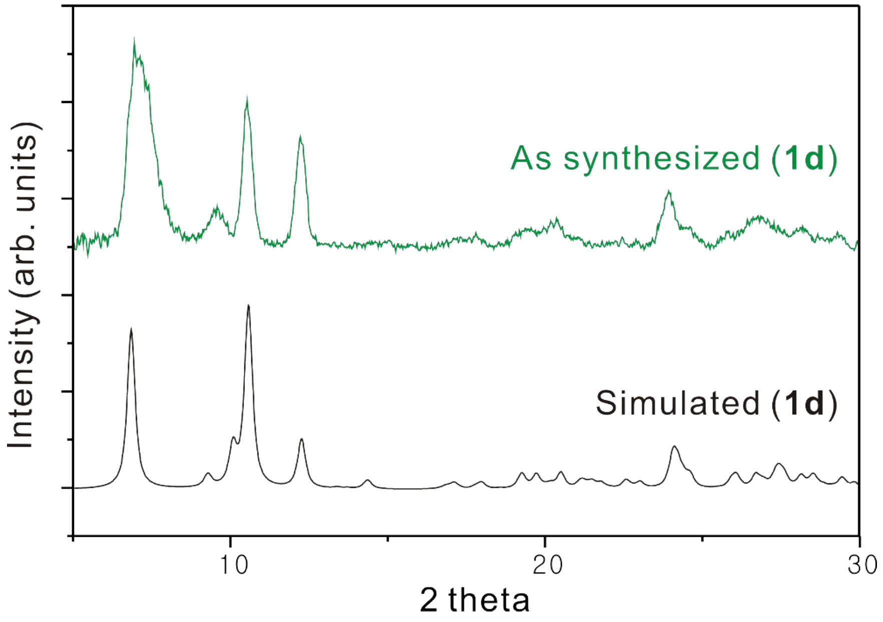

Fig. S7 PXRD patterns for **1d**: (top) as synthesized and (bottom) simulated from the single crystal X-ray data.

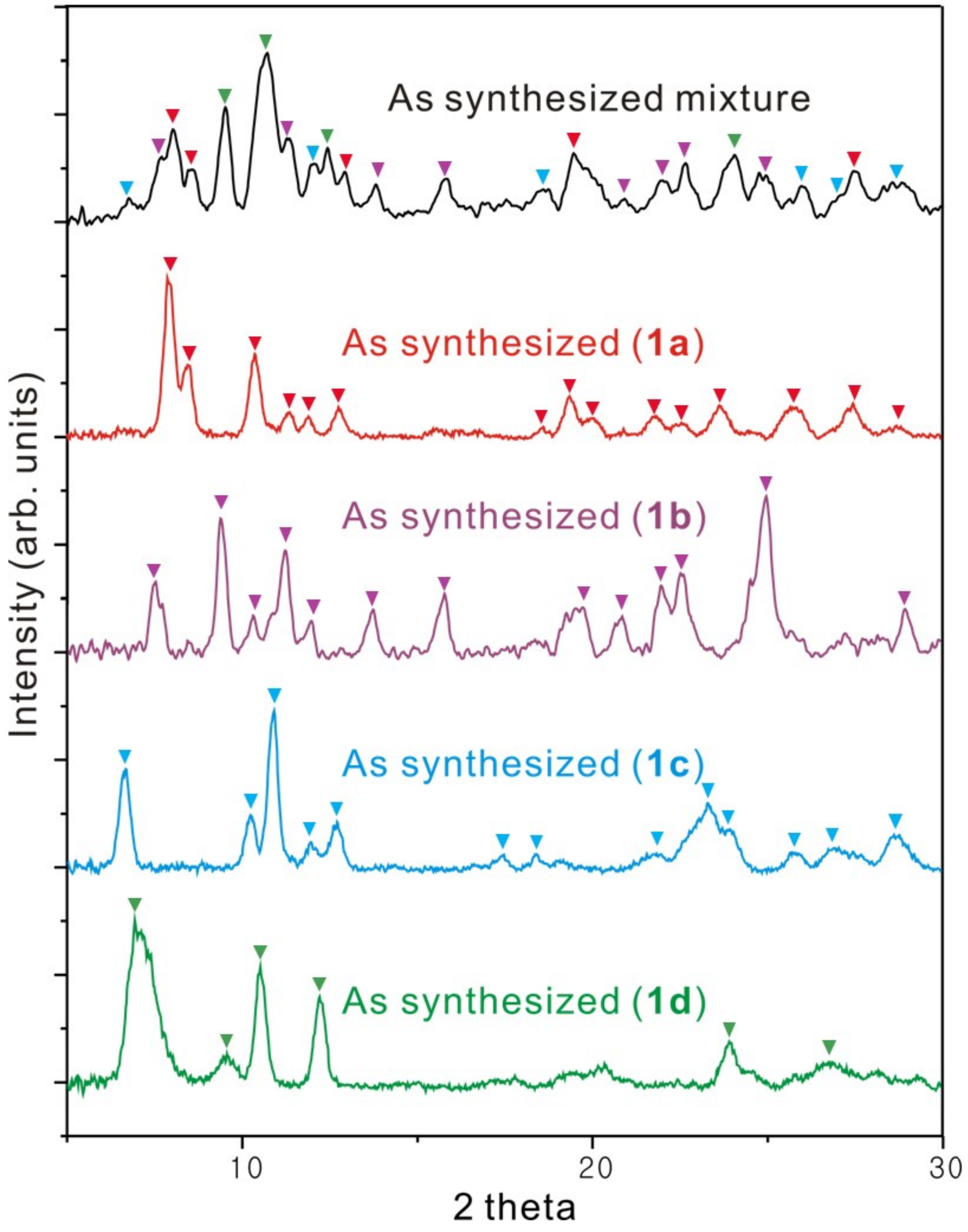

Fig. S8 Comparison of PXRD patterns for the mixed products (**1a**-**1d**) and each separated product.

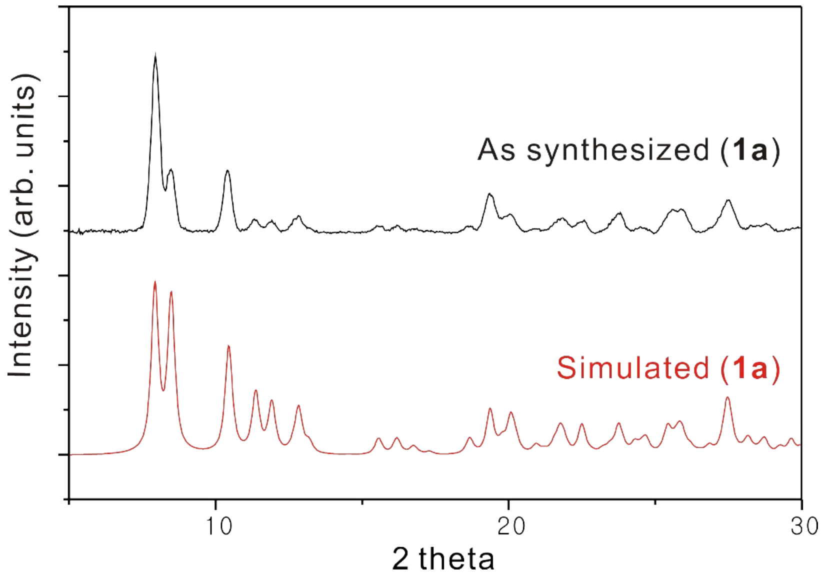

Fig. S9 PXRD patterns for **1a** obtained from stirring the reaction mixture: (top) as synthesized and (bottom) simulated from the single crystal X-ray data. (*Comment*)**:** Unlike the formation of the crystalline mixed products, the solid powder product obtained after stirring the reaction mixture of **L** and CuI for 30 min was confirmed to be of type **1a** by its PXRD pattern. As a result, we were not able to obtain the required pure crystalline CuI complex of **L** by using a direct synthetic approach.

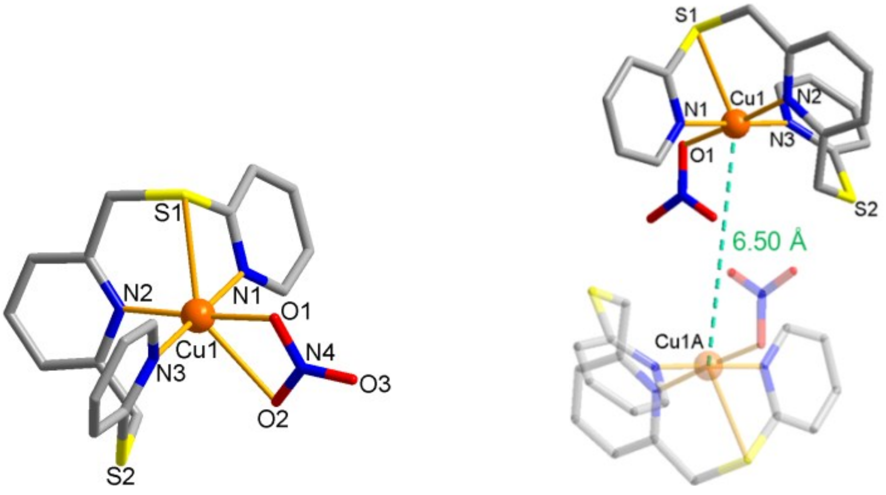

(a) (b)

Fig. S10 Copper(II) nitrate complex [Cu^{II}(**L**)NO_3]NO_3·toluene (**2**): (a) perspective view of mononuclear complex and (b) view of two nearest complex units showing a cooper-to-copper distance 6.50 Å. Non-coordinating anion and solvent molecule are omitted. A) 0.5-x, 0.5-y, 1-z

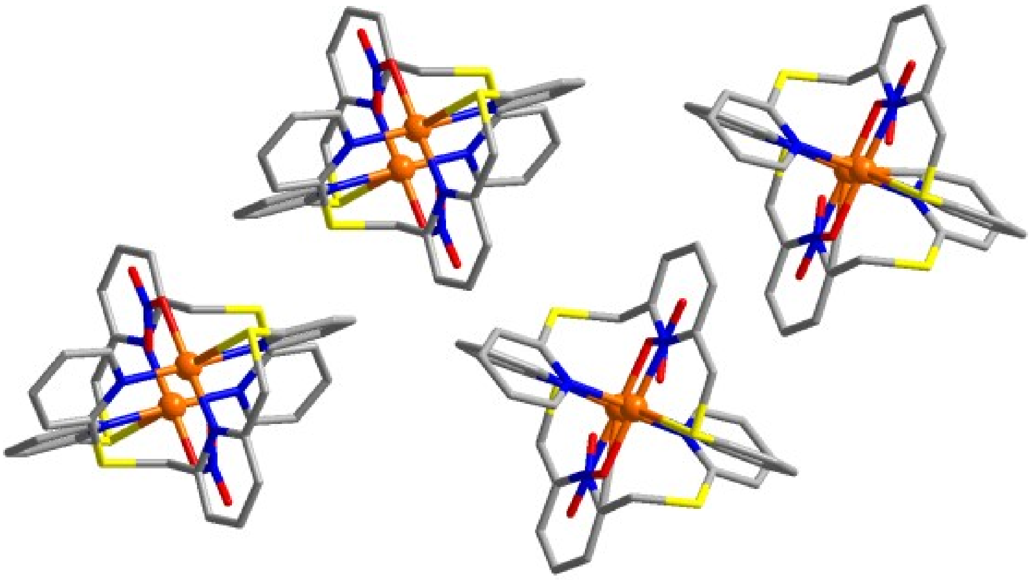

(a)

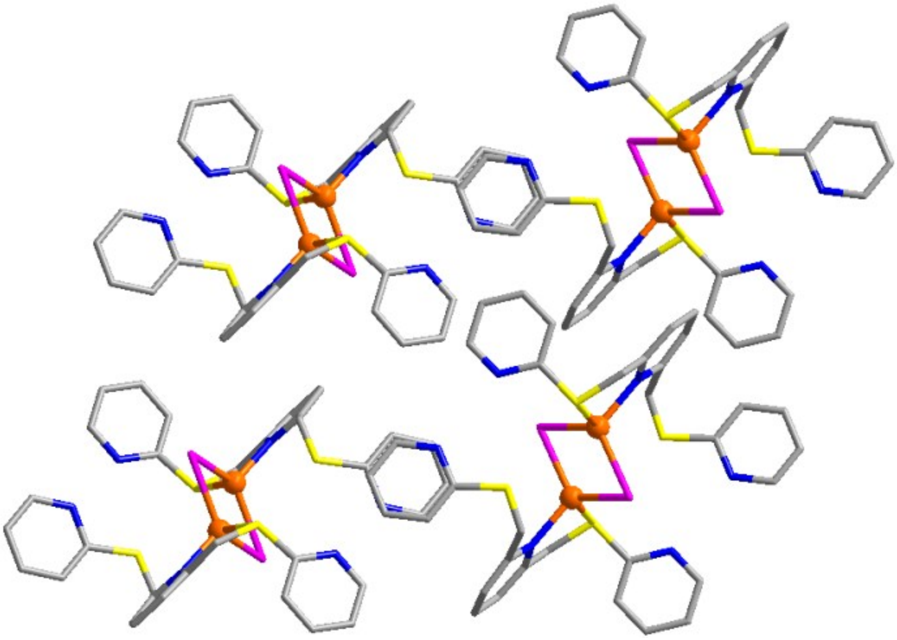

(b)

Fig. S11 Packing structures of (a) **2** and (b) **3**.

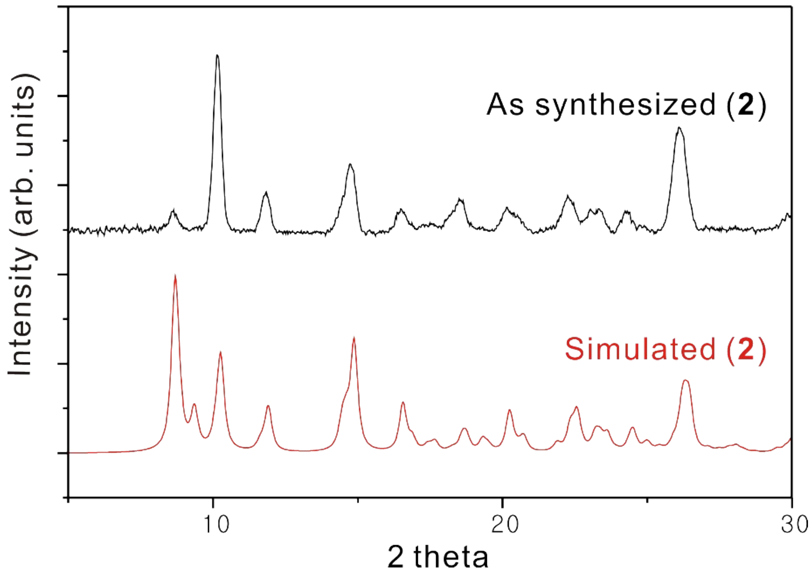

Fig. S12 PXRD patterns for **2**: (top) as synthesized and (bottom) simulated from the single crystal X-ray data.

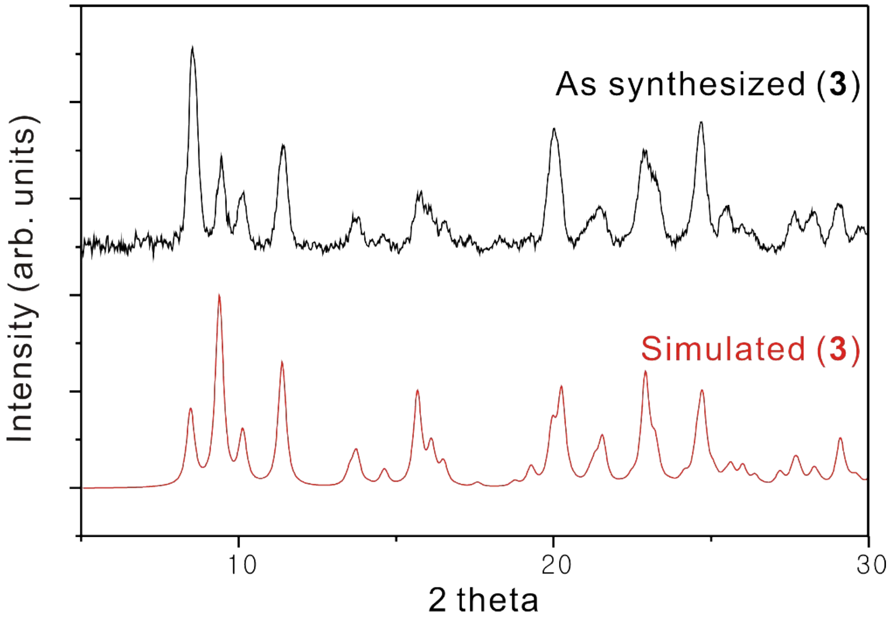

Fig. S13 PXRD patterns for **3**: (top) as synthesized and (bottom) simulated from the single crystal X-ray data.

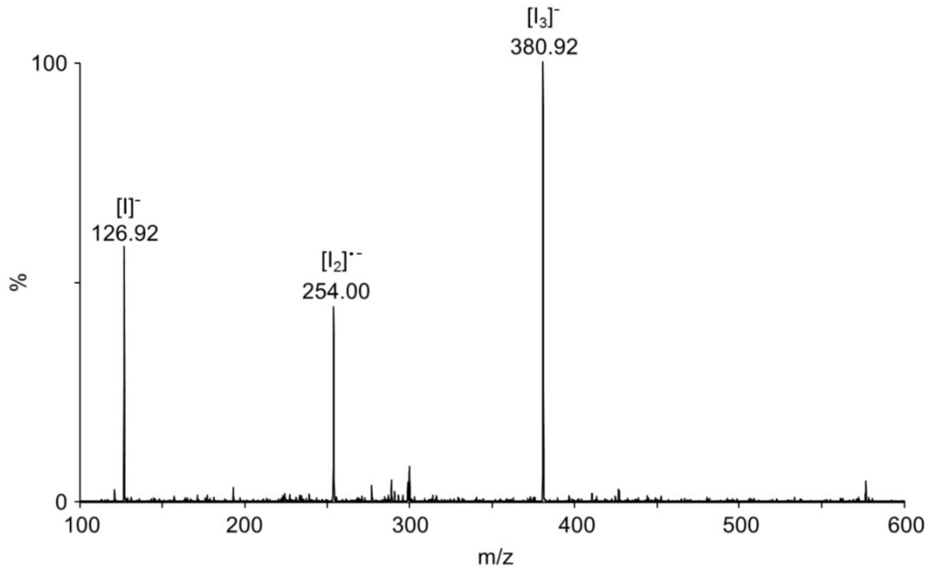

Fig. S14 ESI-MS spectrum of supernatant NaI aqueous solution containing single crystals of **2**.

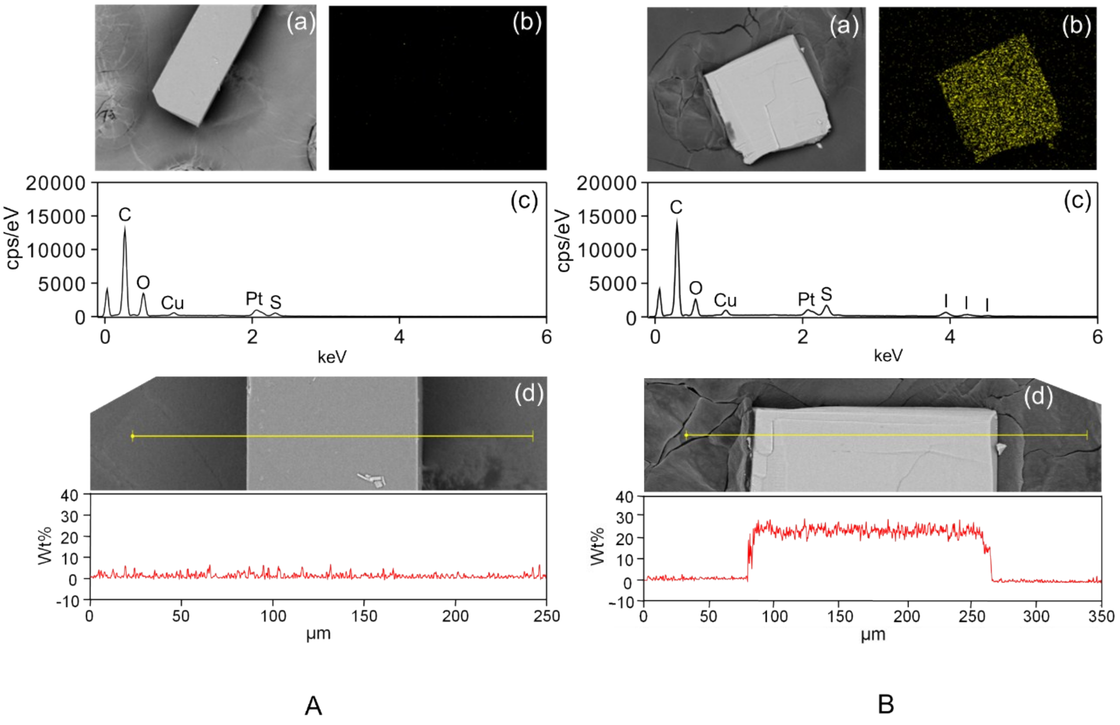

Fig. S15 SEM image and EDS line mapping for (A) **2** and (B) **3**.

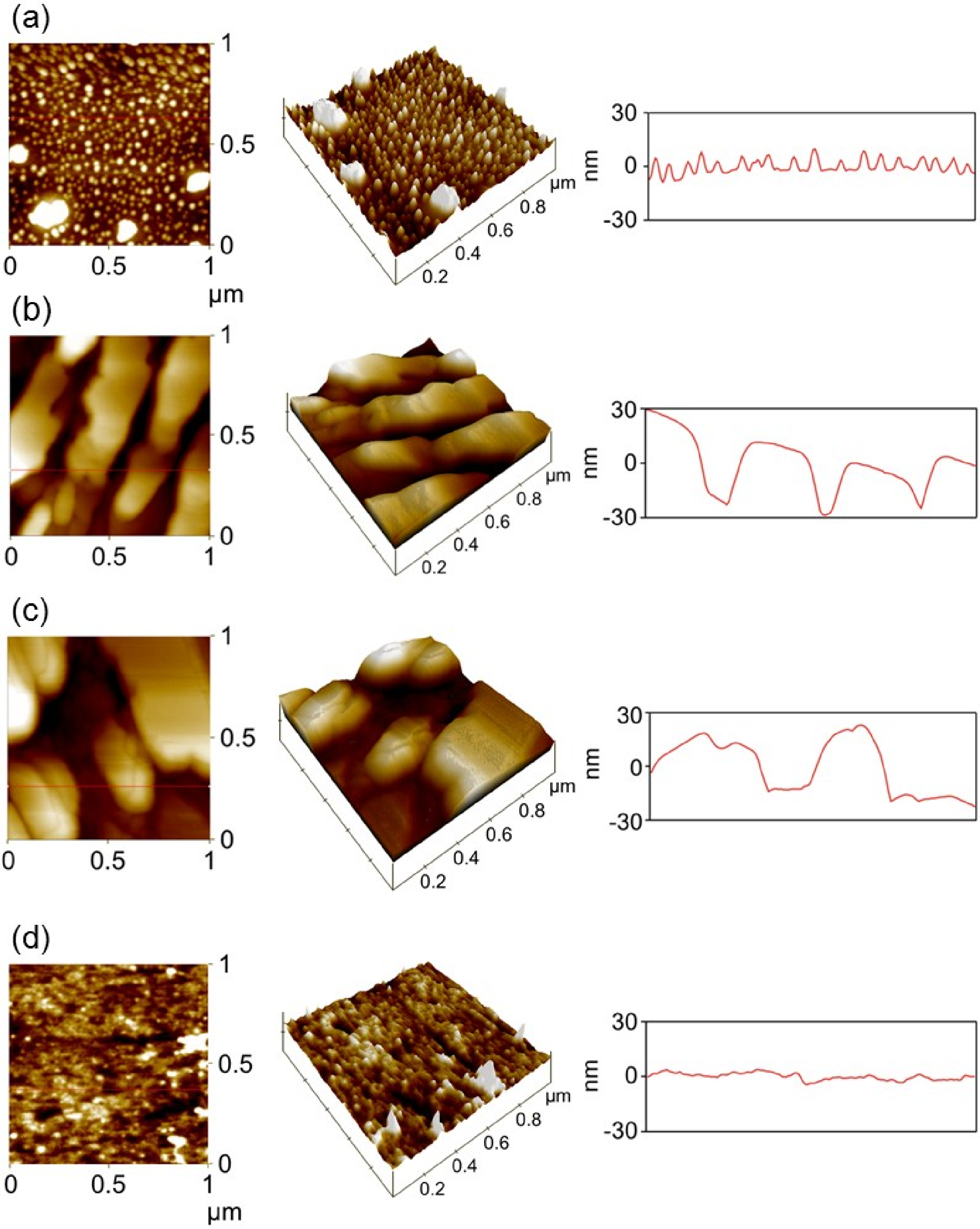

Fig. S16 AFM images and profiles of the surface of a single crystal of **2** before and after immersing in 3 M NaI aqueous solution: (a) before anion exchange, (b) 24 h, (c) 48 h and (d) 72 h.

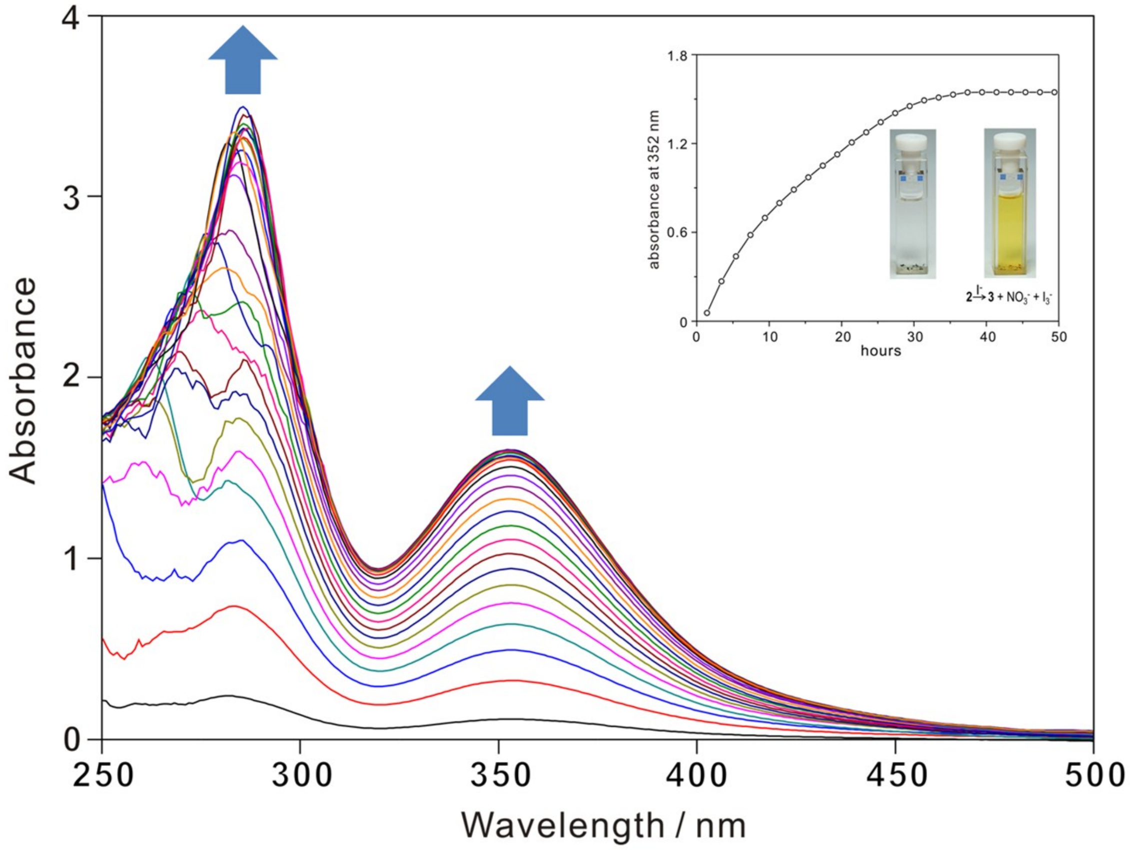

Fig. S17 Time-dependent releasing profile of NO_3^- release from **2** by detecting I_3^- in the supernatant NaI aqueous solution.

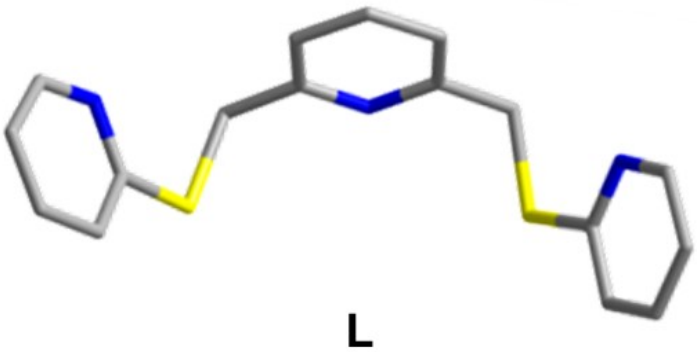

L

Fig. S18 Crystal structure of **L**.

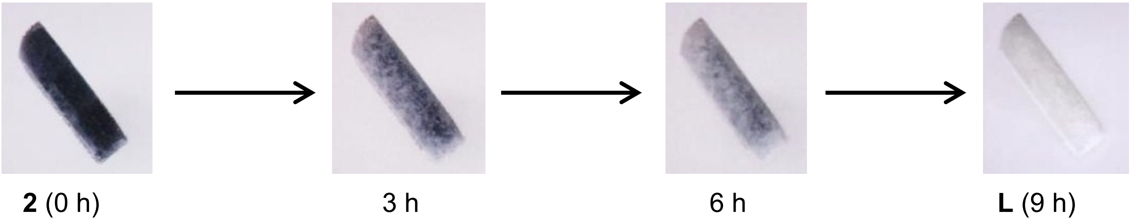

Fig. S19 Photographs of a single crystal of **2** before and after immersing in 3 M NaCl aqueous solution or water.

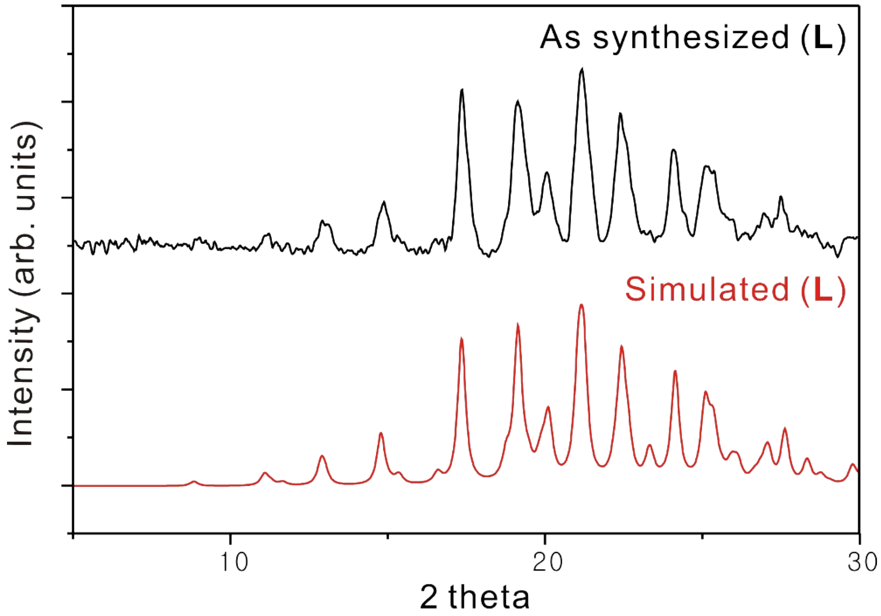

Fig. S20 PXRD patterns for **L**: (top) as synthesized and (bottom) simulated from the single crystal X-ray data.

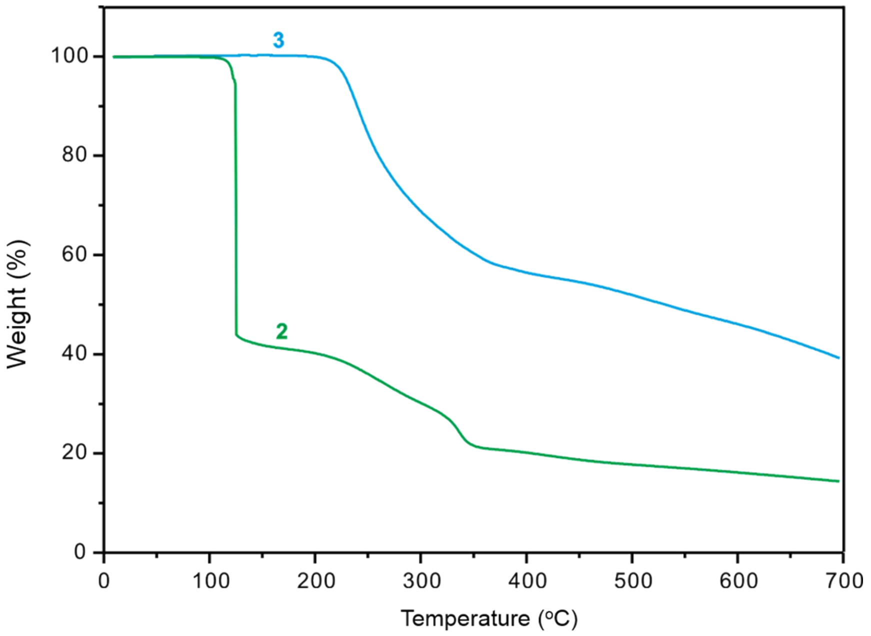

Fig. S21 TGA curves for **2** and **3** with heating rate of 5 °C·min^{-1} under N_2 flow.

Table S1 Crystal and experimental data for **1a-1d**

	**1a**	**1b**	**1c**	**1d**
Formula	$C_{34}H_{30}Cu_4I_4N_6S_4$	$C_{34}H_{30}Cu_4I_4N_6S_4$	$C_{35}H_{32}Cl_2Cu_4I_4N_6S_4$	$C_{36}H_{34}Cl_4Cu_4I_4N_6S_4$
Formula weight	1412.64	1412.64	1497.57	1582.49
Temperature	173(2)	173(2)	173(2)	173(2)
Crystal system	Monoclinic	Triclinic	Monoclinic	Monoclinic
Space group	$P2_1/n$	P-1	$C2/c$	$C2/c$
Z	2	1	4	4
a /Å	14.0030(3)	8.81100(10)	28.2650(8)	25.9718(6)
b /Å	8.7535(2)	10.09970(10)	16.1159(5)	10.2757(2)
c /Å	16.9635(3)	12.5440(2)	10.1658(3)	17.5936(4)
α /deg	90	101.5230(10)	90	90
β /deg	93.9690(10)	102.9950(10)	109.4630(10)	90.9400(10)
γ /deg	90	104.2830(10)	90	90
V /Å^3	2074.32(7)	1014.82(2)	4366.1(2)	4694.72(18)
D_{calc} /(g/cm^3)	2.262	2.311	2.278	2.242
$2\theta_{max}$ **(°)**	52	52	52	52
R_1, wR_2 [$I > 2\sigma(I)$]	0.0168, 0.0415	0.0171, 0.0438	0.0306, 0.0774	0.0464, 0.1163
R_1, wR_2 [all data]	0.0186, 0.0422	0.0184, 0.0445	0.0336, 0.0801	0.0508, 0.1194
Goodness-of-fit on F^2	1.036	1.144	1.047	1.101
No. of reflns used [>2σ(I)]	4079 [R_{int} = 0.0254]	3994 [R_{int} = 0.0224]	4306 [R_{int} = 0.0449]	4613 [R_{int} = 0.0340]
Structure determination	SHELXTL	SHELXTL	SHELXTL	SHELXTL
Refinement	full-matrix	full-matrix	full-matrix	full-matrix

Table S2 Crystal and experimental data for **2**, **3** and **L**

	**2**[a]	**3**	**L**
Formula	$C_{17}H_{15}CuN_5O_6S_2$	$C_{34}H_{30}Cu_2I_2N_6S_4$	$C_{17}H_{15}N_3S_2$
Formula weight	513.00	1031.76	325.44
Temperature	173(2)	173(2)	173(2)
Crystal system	Monoclinic	Monoclinic	Monoclinic
Space group	*C2/c*	$P2_1$/n	$P2_1$/n
Z	4	2	4
a (Å)	17.88900(10)	10.1355(2)	10.5537(5)
b (Å)	12.23390(10)	8.3969(2)	9.2526(4)
c (Å)	20.7705(3)	21.0712(4)	16.3350(8)
α (°)	90	90	90
β (°)	101.7030(10)	95.5800(10)	107.345(3)
γ (°)	90	90	90
V (Å^3)	4451.18(8)	1784.80(6)	1522.56(12)
D_{calc} (g/cm^3)	1.618	1.920	1.420
$2\theta_{max}$ (°)	52.00	52.00	52.00
R_1, wR_2 [$I > 2\sigma(I)$]	0.0251, 0.0645	0.0904, 0.1791	0.0506, 0.0822
R_1, wR_2 [all data]	0.0280, 0.0655	0.1080, 0.1920	0.0864, 0.0919
Goodness-of-fit on F^2	1.071	1.054	1.074
No. of reflection used [>2σ(I)]	4370 [R_{int} = 0.0289]	3508 [R_{int} = 0.0531]	2981 [R_{int} = 0.0558]
Structure determination	SHELXTL	SHELXTL	SHELXTL
Refinement	full-matrix	full-matrix	full-matrix

[a] Since the lattice solvent molecules in **2** are highly disordered, the contribution of solvent electron density was removed by the SQUEEZE routine in PLATON.[S1]
